# Immunogenicity of *Escherichia coli* Outer Membrane Vesicles: Elucidation of Humoral Responses against OMV-Associated Antigens

**DOI:** 10.3390/membranes13110882

**Published:** 2023-11-16

**Authors:** Lorenzo Croia, Giulia Boscato Sopetto, Ilaria Zanella, Elena Caproni, Assunta Gagliardi, Silvia Tamburini, Enrico König, Mattia Benedet, Gabriele Di Lascio, Riccardo Corbellari, Alberto Grandi, Michele Tomasi, Guido Grandi

**Affiliations:** 1 Department of Cellular, Computational and Integrative Biology (CIBIO), University of Trento, Via Sommarive 9, 38123 Trento, Italy; lorenzo.croia@unitn.it (L.C.); g.boscatosopetto@unitn.it (G.B.S.); ilaria.zanella@unitn.it (I.Z.); koenig.enrico@gmail.com (E.K.); riccardo.corbellari@unitn.it (R.C.); michele.tomasi.2@unitn.it (M.T.); 2 Toscana Life Sciences Foundation, Via Fiorentina 1, 53100 Siena, Italy; e.caproni@toscanalifesciences.org (E.C.); a.gagliardi@toscanalifesciences.org (A.G.); silvia.tamburini@unitn.it (S.T.); m.benedet@toscanalifesciences.org (M.B.); g.dilascio@toscanalifesciences.org (G.D.L.); a.grandi@toscanalifesciences.org (A.G.); 3 BiOMViS Srl, Via Fiorentina 1, 53100 Siena, Italy

**Keywords:** outer membrane vesicles (OMVs), vaccines, humoral immunity, antibody response, heterologous antigens

## Abstract

Outer membrane vesicles (OMVs) produced by Gram-negative bacteria have emerged as a novel and flexible vaccine platform. OMVs can be decorated with foreign antigens and carry potent immunostimulatory components. Therefore, after their purification from the culture supernatant, they are ready to be formulated for vaccine use. It has been extensively demonstrated that immunization with engineered OMVs can elicit excellent antibody responses against the heterologous antigens. However, the definition of the conditions necessary to reach the optimal antibody titers still needs to be investigated. Here, we defined the protein concentrations required to induce antigen-specific antibodies, and the amount of antigen and OMVs necessary and sufficient to elicit saturating levels of antigen-specific antibodies. Since not all antigens can be expressed in OMVs, we also investigated the effectiveness of vaccines in which OMVs and purified antigens are mixed together without using any procedure for their physical association. Our data show that in most of the cases OMV–antigen mixtures are very effective in eliciting antigen-specific antibodies. This is probably due to the capacity of OMVs to “absorb” antigens, establishing sufficiently stable interactions that allow antigen–OMV co-presentation to the same antigen presenting cell. In those cases when antigen–OMV interaction is not sufficiently stable, the addition of alum to the formulation guarantees the elicitation of high titers of antigen-specific antibodies.

## 1. Introduction

Outer membrane vesicles (OMVs) are closed spheroid particles, 50 to 300 nm in diameter, that exert a multitude of key biological functions in Gram-negative bacteria [1,2]. Discovered more than 60 years ago, OMVs have recently gained attention for vaccine applications. They carry many microbe-associated molecular patterns (MAMPs) [3,4,5] that work synergistically [6] and promote Th1/Th17-skewed immune responses [7]. Moreover, OMVs can be decorated with foreign antigens/epitopes by proper genetic manipulation of the OMV-producing strains. Following the pioneer work by Kesty and Kuehn [8], an increasing number of heterologous proteins have been successfully delivered to OMVs using a variety of strategies [6,9,10,11]. Moreover, OMVs can be rapidly and easily purified from the bacterial culture supernatant. The original OMV production methods involve the treatment of bacterial biomass with mild detergents [12], but more recently, detergent-free methods based on hypervesiculating strains have been proposed [9,13,14,15,16]. By using tangential flow filtration (TFF), production yields higher than 100 mg of vesicles per liter of culture have been reported [17].

OMV-based vaccines can be classified into two categories. The first one includes vaccines constituted by OMVs directly purified from the pathogen of interest [14,15]. The rationale for developing such vaccines is that OMVs provide both the immune stimulatory molecules (adjuvants) and the protective antigens. OMV-based vaccines belonging to this category have already reached the market [18,19], while others are in advanced clinical phases [20,21]. The second category includes OMVs decorated with heterologous antigens and purified from a properly engineered Gram-negative bacterium [14,15]. These vaccines exploit OMVs as a delivery and immunostimulatory platform and they have the advantage of potentially targeting any kind of infectious disease. 

No vaccines belonging to this second category are currently available. A potential obstacle for developing such vaccines resides in the OMV “loading capacity”, which may not be sufficient to accommodate enough recombinant antigen to elicit a protective immune response. Such consideration comes from the notion that the amount of antigens included in the adjuvanted subunit-based vaccines normally exceeds 10 μg per dose. For instance, the Bexsero vaccine contains a 50 μg/dose of each of the three protective recombinant proteins and the GSK acellular pertussis vaccine includes 25–25 and 8 μg of pertussis toxin, filamentous hemagglutinin, and pertactin, respectively [22]. Should these quantities of antigens be required to reach comparable immune responses with recombinant OMV-based vaccines, even assuming an antigen expression between 10 and 20% of the total OMV proteins, each vaccine dose should well exceed 100 μg of vesicles. Considering the potent OMV adjuvanticity, this OMV dosage might pose regulatory issues.

Fortunately, the data in animal models indicate that antigen-specific immune responses can be obtained with relatively low doses of recombinant OMVs. For instance, Huang and colleagues showed that 50 μg of engineered *Escherichia coli* DH5α OMVs expressing *Acinetobacter baumannii* Omp22 at 1% of total OMV proteins elicited antibody titers comparable to 50 μg of purified Omp22 + aluminum hydroxide (alum) [23]. Kuipers et al. showed that the intranasal immunization of mice with 4 μg of Salmonella OMVs engineered with two *Streptococcus pneumoniae* proteins induced strong protection in a murine model of pneumococcal colonization [24]. We recently showed that 0.2 μg of *E. coli* OMVs carrying *Staphylococcus aureus* antigens at concentrations ranging from 5 to 20% of total OMV proteins were sufficient to induce saturating levels of antigen-specific antibodies [25]. However, a systematic analysis aimed at establishing the amount of OMVs necessary and sufficient to induce proper antigen-specific antibody responses is missing and has not been published yet. 

The aim of this work was to systematically analyze how the amount of recombinant antigens present in *E. coli* OMVs influences the antigen-specific antibody responses in mice. 

## 2. Materials and Methods

### 2.1. Animal Studies

Female CD1 mice of 6–8 weeks were purchased from Charles River Laboratories (Maiano, Italy). Animal experiments were carried out in accordance with the experimental protocols reviewed and approved (1060/2016-PR and 1153/2020-PR) by the Animal Ethical Committees of the University of Trento (Trento, Italy), Toscana Life Sciences (Siena, Italy), and by the Italian Ministry of Health. Mice were monitored once a day to evaluate any sign of stress such as respiration rate, posture, and loss of weight (more than 20%). Mice showing such conditions were anesthetized and subsequently sacrificed in accordance with the experimental protocols.

For immunogenicity analyses, animals were intraperitoneally (i.p.) immunized three times (days 0-14-28) with different concentrations of OMVs formulated alone or in the presence of 2 mg/mL aluminum hydroxide (alum) (Alhydrogel^®^ 10 mg/mL, Invivogen, San Diego, CA, USA) in a final volume of 200 μL/dose formulated in phosphate-buffered saline (PBS) (Gibco, Thermo Fisher Scientific, Waltham, MA, USA) + 35 g/L NaCl (corresponding to 400 μg of alum/dose). Using the same immunization schedule and protocol, twelve groups of four CD1 mice per group were immunized with 10 µg of the following purified recombinant proteins: CpoB, LpoA, SurA, YncE, FkpA, GlpQ, MalE, HisJ, OppA, BamA, OmpF, and FhuE. Mouse sera were collected 10 days after the last immunization. 

### 2.2. Bacterial Strains and Cultures

The *E. coli* HK100 strain was used for cloning experiments by applying the polymerase incomplete primer extension (PIPE) method [26]. The newly generated plasmids of interest were transformed into the *E. coli* BL21(DE3), *E. coli* BL21(DE3)Δ*ompA*, and *E. coli* BL21(DE3)Δ60 strain [27]. Recombinant strains were grown in lysogeny broth (LB) medium (25 g/L, Sigma, St. Louis, MO, USA) at 30 °C or 37 °C, under agitation (200 rpm), in the presence of 100 μg/mL Ampicillin (Carl Roth, KA, Karlsruhe, Germany) when required. The bacterial strains were grown in flasks (50 mL or 500 mL cultures) or in a 2 L ez-Control bioreactor (Applikon). For fermentation processes, bacteria were grown at 30 °C until OD_600_ = 0.5, then growth continued at 25 °C, pH 6.8 (±0.2), dO2 > 30%; rpm 280–500 rpm. Antigen expression was induced at OD_600_ = 1.0 by adding 0.1 mM isopropyl β-D-1-thiogalactopyranoside (IPTG) (Merk, DA, Darmstadt, Germany) and the required antibiotic at a final concentration of 100 μg/mL. 

### 2.3. Cloning Strategies 

The polymerase incomplete primer extension (PIPE) method was applied to clone in the pET15b+ plasmid the *cpoB, lpoA, pal, rlpA, surA, yncE, fkpA, glpQ, malE, ydcL, hisJ, oppA, ybiS, bamA, ompF, fhuE fhuD2, hla_H35L_, spA_KKAA_, slo*, and *spyCEP* genes fused at the 5′ end to a His6x-TAG coding sequence. I-PIPE amplification products were generated by PCR using the couples of primers reported in Table S2. The plasmid was linearized by PCR (V-PIPE) using GCCCTGGAAGTACAGGTTTTC-F/CGCGACTTAATTCTAGCATAAC-R primers, and a digestion with DpnI (Thermo Fisher Scientific, MA, USA) was performed to eliminate the residual quantity of the circular template plasmid. Linearized vectors and I-PIPE products were co-transformed in the *E. coli* HK100 chemically competent strain and plated on LB-Agar (Fisher bioreagents, Milan, Italy) supplemented with the required antibiotic. The correctness of the sequence of each construct was verified by DNA sequencing using primers CCCTATAGTGAGTCGTATTA-F/GCTAGTTATTGCTCAGCGG-R (Table S3) and analyzed with Benchling software (Benchling, Inc., San Francisco, CA, USA, 2023 https://benchling.com, accessed on 5 October 2023). Finally, the newly generated plasmids (listed in Table S2) were used to transform *E. coli* BL21(DE3). The pET21b(+) plasmid was linearized by PCR (V-PIPE) using the primers EK-1084-F/EK-1085-R (Table S3). The recombinant plasmids were finally used to transform the *E. coli* BL21(DE3)Δ*ompA* and the *E. coli* BL21(DE3)Δ60 strains.

### 2.4. Production and Purification of Recombinant Proteins

Recombinant proteins carrying a N-terminal His6x-tag were purified using the tobacco etch virus protease (TEV) purification strategy. To produce the protein of interest, an overnight culture of each recombinant *E. coli* BL21(DE3) strain (Table 1) was used to inoculate 500 mL of LB containing 100 μg/mL of Ampicillin (Carl Roth, Ka, Germany). The cultures were grown under agitation at 37 °C and at OD_600_ = 0.6, IPTG (0.1 mM) was added, and growths were continued for additional 4 h. Next, the cultures were centrifuged in a Beckman Coulter Avanti^®^ JE Centrifuge (Life Sciences) at 6000× *g* at 4 °C for 20 min. The bacterial biomass from each culture was resuspended in lysing buffer A (50 mM NaH_2_PO_4_, 20 mM NaCl, pH 2.7 ref. Table S1) (10 mL per 1 g of wet weight biomass) in the presence of 0.2 mM phenylmethylsulfonyl fluoride (PMSF, Sigma, MO, USA). Cells were sonicated at 4 °C with a Branson Ultrasonics™ S-450A Model Sonifier™ and centrifuged at 15,000× *g* and 4 °C for 30 min. The supernatant was collected and loaded in an ÄKTA purifier system (GE Healthcare/Amersham Biosciences, Piscataway, NJ, USA) equipped with a 5 mL HiTrap IMAC column (GE Healthcare/Amersham Biosciences, NJ, USA) and equilibrated with buffer A. Protein binding was performed with buffer A and washing of the column with 10% buffer B (Buffer A + 500 mM imidazole). Bound proteins were eluted with an increasing linear gradient of buffer B from 10% to 100% (5 column volumes). All collected fractions (1 mL) were analyzed by SDS-PAGE. Fractions containing the purified His6x-tagged recombinant proteins were pooled and dialyzed against 2 L buffer A using 10 K MWCO SnakeSkin™ Dialysis Tubing (Thermo Fisher Scientific, MA, USA). Dialyzed fractions were digested with TEV (1 mg per 100 mg protein) with the addition of 5 mM β-mercaptoethanol. TEV-digested proteins were applied to Ni-affinity chromatography and the fraction containing the digested recombinant proteins were finally purified by size-exclusion chromatography using a HiLoad 16/600 Superdex 75 pg column (Sigma, St. Louis, MO, USA).

### 2.5. Western Blot and Enzyme-Linked Immunosorbent Assay (ELISA)

Western blot analysis was performed as previously reported [9]. The mouse sera collected 10 days after the third immunization were pooled, diluted 1:1000, and the membranes were incubated overnight at 4 °C. Subsequently, the membranes were washed three times with PBST for 5 min each, prior 1-h incubation with rabbit anti-mouse polyclonal antibody diluted 1:2000 in 3% skimmed milk 0.05% Tween in PBS (PBST). After extensive washing, the WestarNova 2.0 (Cyanagen, Bologna, Italy) chemiluminescent reagent was added and bands revealed using the ChemiDOC (BioRad, Hercules, CA, USA) instrument. ELISA assays on mice sera collected after the immunizations were performed as previously described [9]. Briefly, plates were coated with 200 ng of recombinant protein sera and incubation was performed for 1 h at room temperature. Next, the plates were washed and incubated with alkaline phosphatase (AP)-conjugated, goat anti-mouse IgG (Sigma) for 1 h at room temperature. After washing, 100 μL/well of a solution containing 1 M diethanolamine (DEA), 0.5 mM MgCl_2_, and 3 mg/mL alkaline phosphatase (AP) substrate (Sigma) were added. Plates were incubated at 30 °C for 20 min and absorbance was read at 405 nm using a Varioskan spectrophotometric plate reader. Titers are given as the serum dilution, which gives an OD_4050_ = 2.

### 2.6. OMVs Purification and SDS-PAGE

The recombinant *E. coli* BL21(DE3)Δ*ompA* and *E. coli* BL21(DE3)Δ60 strains were grown in LB medium at 37 °C and 200 rpm agitation starting from an OD_600_ = 0.1. When the cultures reached an OD_600_ = 0.6, antigen expression was induced by the addition of 0.1 mM IPTG. Bacterial growth was continued for four additional hours. Culture supernatants were separated from the biomass and collected by centrifugation at 5000× *g* for 20 min at 4 °C. The supernatants were filtered through a 0.22 μm pore size filter (Merck Millipore, Burlington, MA, USA). OMVs were purified from the supernatants by centrifugation at 200,000× *g* for 2 h using an OptimaTM XE (Beckman Coulter, Brea, CA, USA). The pellets containing the vesicles were resuspended in PBS 1X buffer. OMVs were quantified based on their protein content using the DC™ Protein Assay Kit II (BioRad, CA, USA). The quality of the purified OMVs was verified by SDS-PAGE. Briefly, 10 μg of vesicles were diluted in Laemmli 4X electrophoretic dye (62.5 mM Tris-HCl, pH 6.8 buffer 25% glycerol 2% SDS 0.01% bromophenol blue, 1% dithiothreitol; Bio-Rad, CA, USA), heated at 95 °C for 10 min and loaded on an Any kDTM Criterion^TM^ TGX Stain-Free^TM^ Protein Gel (BioRad, Hercules city, CA, USA). Finally, the OMV protein contents were revealed by Coomassie Blue staining using the ProBlue Safe Stain (Giotto Biotech, Sesto Fiorentino, FI, Italy). 

### 2.7. Software and Data Analysis

**Figure 1:**
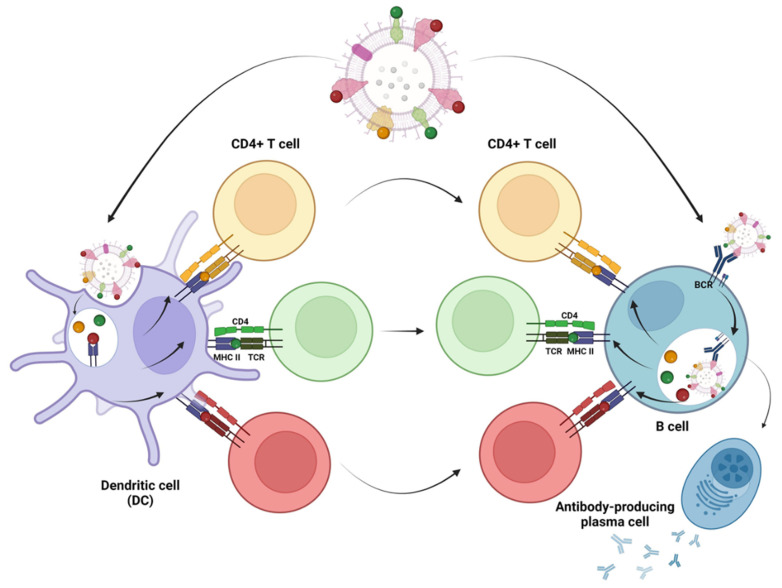
*Proposed role of OMV endogenous proteins in potentiating the humoral responses against heterologous antigens expressed in OMVs*. OMVs are internalized by DCs, which present epitopes from OMV endogenous proteins in the context of the MHC II molecule, thus activating a large population of CD4+ T helper (Th) cells (for simplicity, three T cells recognizing different epitopes are represented with different colors). In parallel, naïve B cells with their receptors recognize the OMV-associated heterologous antigens, and after OMV internalization, present on their MHC II molecules the same OMV-derived epitopes presented by DCs. Consequently, the B cells specific for the heterologous antigens can be activated by the large population of OMV-specific effector T cells, thus becoming memory B cells and plasma cells producing antibodies specific for the heterologous antigens.

Sequence analysis and primer design were performed using the online software Benchling Inc. Western blot images were elaborated and post-processed with Image Lab software version 3.0 (Bio-Rad, Hercules city, CA, USA). Densitometry and SDS-PAGE image analyses were carried out with LI-COR Image Studio^TM^ Lite software version 5.2.5 (LI-COR Biosciences, Lincoln, NE, USA). Graphs and statistical analysis were obtained with GraphPad Prism 5 (GraphPad software Inc., La Jolla, CA, USA). Figure 1 was created using the software Biorender.com (Canada).

## 3. Results

### 3.1. Theoretical Considerations

OMVs carry endogenous proteins, mostly belonging to the outer membrane and periplasmic compartments. For instance, more than 150–200 major proteins have been characterized in OMVs isolated from *E. coli* [28,29]. Endogenous proteins are expected to have two opposite effects on the antibody responses elicited against the heterologous antigens. On the one hand, they should give rise to a large population of OMV-specific B cells that should “compete” with the heterologous antigen, diluting its immunogenicity down to a level that could be below the optimal protective threshold. On the other hand, OMV proteins carry a plethora of MHC II epitopes, which give rise to a large pool of effector helper CD4+ T cells. Such CD4+ T cells should provide synergistic help to any B cell that recognizes an antigen expressed in OMVs (Figure 1). Therefore, it is predictable that endogenous proteins could contribute to the elicitation of antibodies against engineered antigens, even if expressed at relatively low concentrations. 

### 3.2. OMV Immunization Elicits Antibodies against OMV-Associated Proteins Expressed at Concentrations ≥1% of Total OMV Proteins

To define the minimal concentration of OMV proteins necessary to elicit antibodies upon OMV immunization, we selected a pool of OMV endogenous proteins, measured their concentration, and determined the presence of protein-specific antibodies in the sera from OMV-immunized mice. In so doing, we could establish the existence, if any, of a concentration threshold below which OMV proteins are immunologically “silent” and above which they elicit antibody responses. 

Taking advantage of the availability of the proteomic analysis of the OMVs from *E. coli* BL21(Δ*ompA*) [27], we selected 16 proteins belonging to both the luminal and membrane compartments (Table 1). 

**Table 1:** List of the selected OMV endogenous proteins.

Gene	Annotation	Compartment ^1^	Molecular Weight (kDa)
*cpoB*	Cell division coordinator	PP	25.4
*lpoA*	Penicillin-binding protein activator	LP	70
*pal*	Peptidoglycan-associated lipoprotein	LP	16.6
*rlpA*	Endolytic peptidoglycan transglycosylase	LP	35.7
*surA*	Chaperone	PP	45
*yncE*	Uncharacterized protein	PP	35.3
*fkpA*	FKBP-type peptidyl-prolyl cis-trans isomerase	PP	26.2
*glpQ*	Glycerophosphodiester phosphodiesterase	PP	38.2
*malE*	Maltose/maltodextrin-binding periplasmic protein	PP	40.7
*ydcL*	Uncharacterized lipoprotein	LP	22.4
*hisJ*	Histidine-binding periplasmic protein	PP	26.2
*oppA*	Periplasmic oligopeptide-binding protein	PP	58.4
*ybiS*	Probable L,D-transpeptidase	PP	30.8
*bamA*	Outer membrane protein assembly factor	OM	88.4
*ompF*	Outer membrane porine F	OM	37
*fhuE*	FhuE receptor	OM	77.4

^1^ LP: lipoprotein; OM: outer membrane protein; PP: periplasmatic protein.

**Figure 2:**
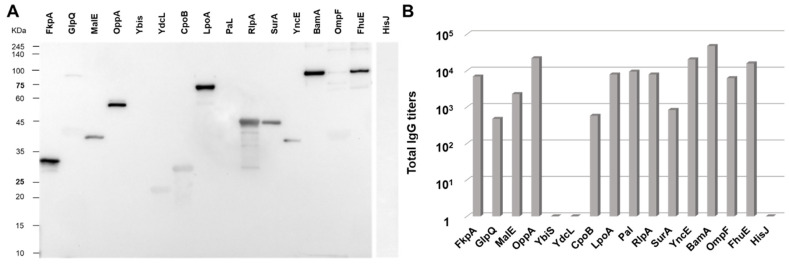
*Immunogenicity of OMV proteins*. Five CD1 female mice were immunized i.p. three times at two-week intervals with 10 μg OMVs_Δ*ompA*_ + alum. Sera were collected 10 days after the third immunization and used to evaluate the presence of antibodies against 16 OMV proteins (Table 1) by Western blot (**A**) and ELISA (**B**) using purified proteins. No error bars are shown, as ELISA was performed in the pooled sera from four mice.

The proteins were expressed in *E. coli* and purified using a two-step affinity chromatography procedure (Figure S1). Next, mice were immunized with 10 μg of OMVs_Δ*ompA*_ + 2 mg/mL of alum and the presence of serum antibodies against the 16 endogenous proteins was assessed by Western blot after electrophoretic separation of the purified proteins. As shown in Figure 2A, the anti-OMVs_Δ*ompA*_ serum recognized nine out of the sixteen purified recombinant proteins. 

Three out of the remaining seven proteins, YdcL, CpoB, and OmpF, were also recognized, but the band intensities were not sufficient to exclude non-specific recognition. The remaining four proteins, GlpQ, Ybis, Pal, and HisJ, were not visible in the Western blot. To further confirm the immunogenicity of the selected proteins, an ELISA was carried out using the purified proteins to coat the plates. As reported in Figure 2B, the Western blot-positive proteins were also all immunogenic by ELISA. Appreciable levels of antibodies were also measured against four additional proteins whereas, consistent with the Western blot data, no titers were measured against YbiS, HisJ, and YdcL. 

**Figure 3:**
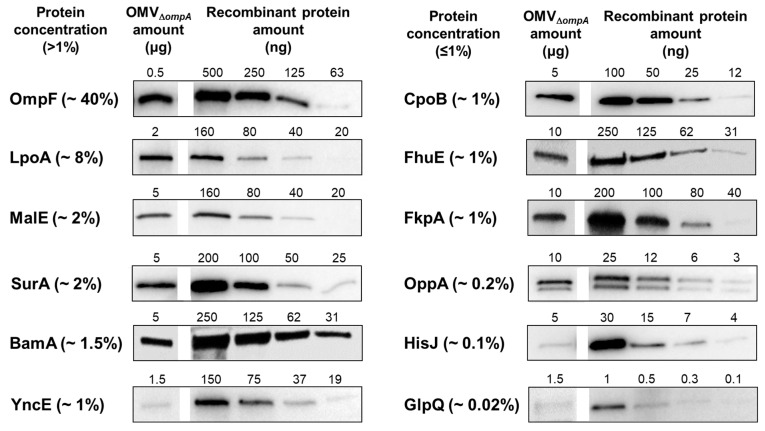
*Quantification of OMV endogenous proteins by semi-quantitative Western blot*. Different quantities of OMVs and purified recombinant proteins were separated by SDS-PAGE, transferred to nitrocellulose membranes, and each protein was visualized using sera collected from mice immunized with the corresponding purified protein. The percentage represents the estimated concentration of the protein relative to the total protein in the OMVs.

Having demonstrated that OMV immunization elicited antibodies against several, but not all OMV proteins, we investigated the expression level of each selected protein in the OMVs by Western blot. Different quantities of each purified protein were run on a polyacrylamide gel together with fixed quantities of OMVs. The separated proteins were transferred onto a nitrocellulose membrane and the proteins were visualized with sera from mice immunized with each purified recombinant protein (Figure 3). By comparing the band intensities between the OMV samples and the purified proteins, we estimated the amount of each protein present in the OMVs (Figure 3 and Table 2). 

The quantification analysis indicated that all proteins classified as immunogenic based on both Western blot and ELISA were present in the OMVs at concentrations >1%. Therefore, it is reasonable to conclude that OMV immunization can elicit measurable levels of antibodies against the proteins present, on average, at concentrations higher than 1%. This is not to say that proteins present at lower amounts may not induce specific antibodies. For example, OppA, which accounted for only 0.2% of total OMV proteins, was also found to be immunogenic. However, HisJ, expressed at an estimated concentration of 0.1%, was negative in both the Western blot and ELISA assays, and therefore, the immunogenicity of poorly expressed proteins (<1%) may vary depending upon their “intrinsic” immunogenicity properties. Protein compartmentalization in the OMV lumen could also have an influence on protein immunogenicity. However, we have previously shown that OMV immunization can elicit high antibody titers against luminal proteins [9]. This is possibly due to the fact that at the site of injection, the vesicles are partially destroyed, thus releasing their content, which becomes visible to the immune system. 

The outer membrane protein OmpF deserves one comment. OmpF is the most abundant protein in OMVs_Δ*ompA*_. However, when the anti-OMVs_Δ*ompA*_ serum was used, OmpF was barely seen in the Western blot, while it appeared to be immunogenic in ELISA. This is likely due to the fact that the OmpF-specific antibodies elicited by OMVs_Δ*ompA*_ immunization mostly recognize the properly folded protein embedded in the membrane, and therefore a fully denatured protein (as it is OmpF when separated by acrylamide gel) is expected to be poorly visible to conformational antibodies. 

### 3.3. A Total of 5 μg of OMVs Are Generally Sufficient to Elicit Plateau Levels of Antibodies against OMV Proteins Expressed at Concentrations ≥2%

**Figure 4:**
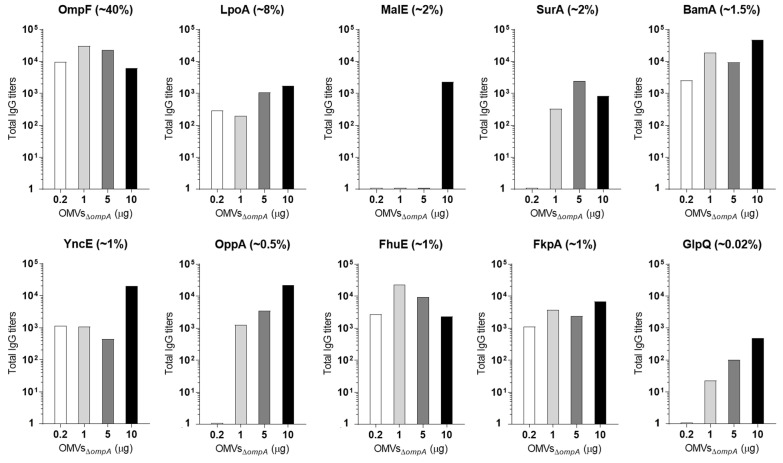
*Protein specific IgG titers in mice immunized with increasing doses of OMVs_ΔompA_.* Groups of CD1 female mice (four mice per group) were immunized three times at 2-week intervals with 0.2 μg, 1 μg, 5 μg, or 10 μg of OMVs_Δ*ompA*_ + alum. Ten days after, the last immunization sera were collected and the IgG titers were analyzed by ELISA using plates coated with purified recombinant proteins. No error bars are shown as ELISA was performed in pooled sera from four mice.

Next, we investigated how immunization with different doses of OMVs could affect the antibody titers against the OMV proteins. Mice were immunized with escalating amounts of OMVs and the antibody titers against the 10 proteins of known concentration in the OMVs and positive in ELISA (Table 2) were measured. More specifically, CD1 mice were immunized three times with 0.2 μg, 1 μg, 5 μg, and 10 μg of OMVs_Δ*ompA*_, and ELISA titers were measured 10 days after the last immunization. The data reported in Figure 4 show that, with the only exception for the periplasmic protein MalE, 5 μg of OMVs were sufficient to elicit high levels of antibodies against the four proteins OmpF, LpoA, BamA, and SurA expressed at concentrations ≥2%. In contrast, 10 μg of OMVs were necessary to induce a sufficiently high level of antibodies against proteins expressed below 1% concentrations. 

**Figure 5:**
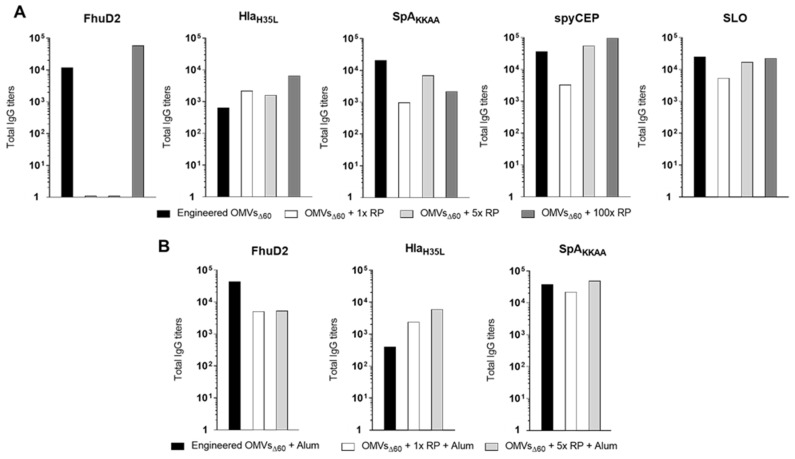
*IgG titers in mice immunized with OMVs_Δ60_ expressing heterologous antigens or with OMVs_Δ60_–antigen mixtures.* (**A**) Groups of CD1 female mice (four per group) were i.p. immunized three times at two-week intervals with 1 μg of engineered OMVs_Δ60_ expressing FhuD2, SpA_KKAA_, Hla_H35L_, SLO, or spyCEP or with 1 μg OMVs_Δ60_ mixed with 1×, 5×, and 100× the amounts of antigens present in the engineered OMVs. Ten days after the last immunization, sera were collected and IgG titers in the pooled sera were analyzed by ELISA using plates coated with the corresponding recombinant antigens. (**B**) The same immunization schedule was performed in the presence of alum, which was added to 1 μg of the engineered OMVs expressing the FhuD2, SpA_KKAA_, or Hla_H35L_ antigens and to 1 μg OMVs_Δ60_ + 5× the amount of antigens present in the engineered OMVs. The antigen-specific ELISA titers were determined as described above. RP, recombinant protein. No error bars are shown as ELISA was performed in pooled sera from four mice.

To further support the conclusion that low quantities of OMVs are sufficient to elicit potent antibody responses against proteins, we selected five recombinant OMVs, each expressing one heterologous antigen from *Staphylococcus aureus* (Hla_H35L_, FhuD2, and SpA_KKAA_) [30,31,32] and *Streptococcus pyogenes* (SpyCEP and SLO) [33,34]. These proteins were expressed in the OMVs at concentrations between 5 and 28% of total proteins (Figure S2). Groups of four mice were immunized three times, two weeks apart, with 1 μg of one of the five engineered OMVs, and after 10 days from the last immunization, the sera from each group were collected, pooled, and antigen-specific antibody titers were measured by ELISA (Figure S3). As shown in Figure 5 (black bars), and consistently with what has been previously published [25], 1 μg of engineered OMVs was sufficient to elicit high antibody titers against all of the recombinant antigens. 

From these data, we can conclude that 5 μg/dose of OMVs is sufficient to elicit saturating levels of antibodies against antigens, which represent >1.5–2% of the total OMV proteins. Some exceptions to this general rule are obviously expected, as is the case for MalE, which, unexpectedly, elicited no antibodies at OMV dosages up to 5 μg and became immunogenic only when 10 μg of OMVs was used. 

### 3.4. Comparison between Antigen-Specific Antibodies Elicited by Engineered OMVs and OMVs + Antigen Mixtures

Not all proteins can be successfully compartmentalized in OMVs. Glycosylated eukaryotic proteins are poorly expressed in bacteria in soluble and functional form, and they hardly reach the periplasmic and outer membrane compartments. Nevertheless, OMVs as an adjuvant are still an attractive option to develop vaccines based on difficult-to-express eukaryotic antigens: they can be produced using appropriate modalities and subsequently mixed with OMVs. However, from a theoretical standpoint, the antigen–adjuvant physical association is a prerequisite to obtain an optimal immune response since antigen presenting cells should simultaneously take up both the antigen and adjuvant [35]. Therefore, a question that remains to be fully addressed is how much antigen needs to be mixed to OMVs to guarantee the induction of high levels of antigen-specific antibodies. 

To answer this question, we immunized mice three times with 1 μg of engineered OMVs expressing either FhuD2, Hla_H35L_, SpA_KKAA_, SLO, or spyCEP, in the absence of alum. In parallel, five groups of mice were immunized with 1 μg of OMVs_Δ60_ mixed with 0.28, 0.1, 0.15, 0.19, and 0.05 μg of recombinant purified FhuD2, Hla_H35L_, SpA_KKAA_, SLO, and spyCEP, respectively. Such dosages (1xRP) correspond to the amounts of each protein present in 1 μg of the corresponding engineered OMVs, as determined by densitometric analysis. Ten days after the third immunization, the blood from animals of each group was collected and antibody titers against each antigen were determined by ELISA using pooled sera as well as single sera from three mice of each group. As shown in Figure 5A and Figure S4, Hla_H35L_- and SLO-specific antibody titers from mice immunized with Hla_H35L_-OMVs_Δ60_ and SLO-OMVs_Δ60_ were essentially superimposable to the titers measured in mice immunized with the OMVs_Δ60_ + 1xRP Hla_H35L_ and OMVs_Δ60_ + 1xRP SLO mixtures. 

The immunizations with OMVs_Δ60_ + 1xRP SpA_KKAA_ and OMVs_Δ60_ + 1xRP spyCEP mixtures induced antibody titers approximately 10 times lower than the titers induced by engineered SpA_KKAA_-OMVs_Δ60_ and SpyCEP-OMVs_Δ60_, respectively. Finally, the formulation of OMVs_Δ60_ + 1xRP FhuD2 did not elicit appreciable levels of anti-FhuD2 antibodies. Based on these data, mice were immunized with OMV–protein mixtures containing five-fold higher amounts of FhuD2, SpA_KKAA_, and SpyCEP (1.4, 0.5, and 0.75 μg, respectively) (5xRP). Titers increased in the case of the OMVs_Δ60_ + 5xRP SpA_KKAA_ and OMVs_Δ60_ + 5xRP Hla_H35L_ mixtures, while they remained low with the OMVs_Δ60_ + 5xRP FhuD2 mixture. Only when 28 μg of purified FhuD2 was added to OMVs_Δ60_ (a FhuD2 concentration 100-fold higher than what was present in FhuD2-OMVs_Δ60)_ (100xRP)) were titers comparable to those obtained with FhuD2-OMVs_Δ60_ measured (Figure 5A).

### 3.5. The OMV–Alum Combination Guarantees High Immunogenicity of Any OMV–Antigen Mixture 

A plausible interpretation of the data described above is that four out of the five tested antigens established an interaction with the OMVs sufficiently stable to be taken up by the antigen-presenting cells together with the vesicles. In contrast, the physical-chemical properties of FhuD2 prevent its association to OMVs, and therefore only in the presence of high FhuD2 concentrations can antigen–adjuvant co-delivery to DCs occur. To support this hypothesis, immunizations with 1 μg of OMVs_Δ60_ mixed with 0.28, 0.1, and 0.15 μg of FhuD2, Hla_H35L_, and SpA_KKAA_, respectively, were repeated in the presence of 2 mg/mL of alum. Alum forms particulates, which are likely to absorb both OMVs and protein antigens. When antigen-specific antibody titers were compared to those obtained with 1 μg of engineered OMVs, similar titers were measured for all three antigens (compare the white bars in Figure 5B with the black bars in Figure 5A). When recombinant proteins were mixed to OMVs at a concentration five-fold higher than that present in 1 μg of recombinant OMVs, a further slight increase in total IgGs was observed. Such an increase was also partially detected when immunization with recombinant FhuD2-OMVs and SpA-OMVs was carried out in the presence of alum (compare the black bars in Figure 5A,B). 

**Figure 6:**
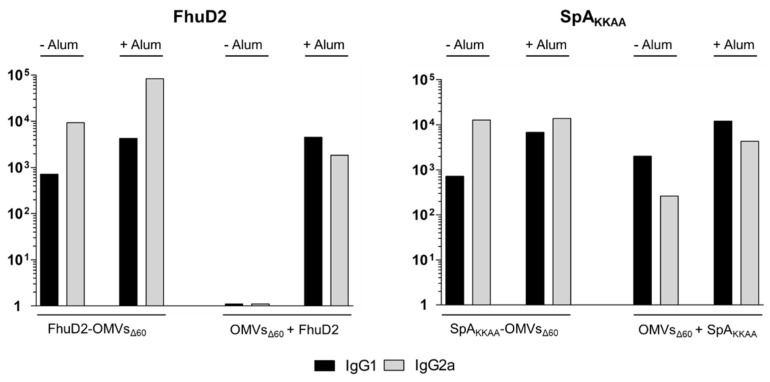
*Effect of the addition of alum on the antibody isotype profile of OMVs_Δ60_ immunization***.** Groups of four CD1 mice were i.p. immunized three times at two week intervals with 1 μg FhuD2-OMVs_Δ60_ and SpA_KKAA_-OMVs_Δ60_ or with 1 μg OMVs_Δ60_ mixed with 0.28 μg of FhuD2 or with 0.15 μg of SpA_KKAA_ in the presence or absence of 2 mg/mL of aluminum hydroxide. Ten days after, the third immunization sera were collected, and pooled, and the IgG1 and IgG2a titers were evaluated by ELISA. No error bars are shown as ELISA was performed in pooled sera from four mice.

Alum is known to skew a Th2-type of response. Therefore, the addition of alum might alter the Th1/Th17 profile of OMV adjuvanticity. To shed light on the possible effect of the addition of alum to the immune response profile of OMVs, the IgG2a and IgG1 titers were assessed by ELISA in the sera from mice immunized with 1 μg of OMVs_Δ60_ mixed with 0.28 μg of RP FhuD2 or 0.15 μg of RP SpA_KKAA_ antigens, in the presence or absence of 2 mg/mL of alum. As shown in Figure 6, the addition of alum to FhuD2-OMVs increased the titers of both IgG2a and IgG1. In the case of SpA_KKAA_-OMVs, alum did not substantially alter the levels of IgG2a and marginally increased the IgG1 titers. The addition of alum to OMVs + protein mixtures elicited IgG2a titers similar to those obtained with the engineered OMVs in the absence of alum, while those for IgG1 were slightly increased.

In conclusion, thanks to the OMV_Δ60_ immunogenicity and capacity to “absorb” most of the tested antigens, OMV–antigen mixtures containing 1 μg of OMVs_Δ60_ + 1 μg of antigen are expected to be sufficient to reach the plateau of antigen-specific antibody titers. The addition of alum to 1 μg of OMVs_Δ60_ + 1 μg of antigen guaranteed “saturating” antibody titers for all of the tested OMV–antigen mixtures. Moreover, the addition of alum did not substantially alter the Th1 profile characterizing the immune responses of the OMV-containing formulations.

## 4. Discussion

OMV immunization elicits humoral responses against OMV-associated proteins. Such responses can be so effective that vaccines based on OMVs purified from pathogenic Gram-negative bacteria have already reached the market or are in advanced clinical studies [19,27,36,37,38,39,40]. However, which of the OMV proteins are either immunogenic or immunologically silent and how proteins either compete or synergize with each other remains elusive. 

With this work, we have filled this knowledge gap by analyzing the antibody responses against proteins present in *E. coli* OMVs. We selected 16 proteins localized either in the lumen or in the membrane of OMVs and we showed that proteins that are expressed in the OMVs at concentrations higher than 1% elicit specific antibody responses when mice are immunized with 10 μg/dose of OMVs. 

Moreover, we showed that as little as 1 to 5 μg of OMVs was sufficient to induce saturating levels of antibodies against most proteins present in the OMVs at concentrations higher than 1.5–2%. This was true for both the endogenous OMV proteins and for different recombinant proteins expressed in the OMVs [25]. Considering that the strong adjuvanticity of OMVs might lead to reactogenic responses if administered above certain threshold levels, these data are particularly relevant for vaccine design. As a general rule, they indicate that minute amounts of recombinant *E. coli* OMVs are necessary and sufficient to induce effective immune responses.

As already pointed out, not all proteins can be successfully compartmentalized in OMVs. For instance, glycosylated eukaryotic proteins are poorly expressed in bacteria, and they hardly reach the OMV compartment. A second important message of this work is the demonstration that OMVs can effectively be used as an adjuvant for vaccines based on recombinant proteins. It is known that the co-delivery of antigen and stimulatory molecules to the same APC is necessary to elicit optimal T-dependent immune responses. Based on such theoretical considerations, which dictate that the adjuvant and antigen should be physically associated to elicit protective immune responses, elegant strategies have been devised to bind purified antigens to OMVs [9,25,41]. However, our data show that such strategies are not necessarily required, and that the simple mixing of OMVs with an antigen can be sufficient to induce effective immune responses. In most of the cases we tested, elevated antigen-specific antibody titers could be obtained following immunization with OMVs mixed with the purified antigens. To reconcile this result with immunological theory, we believe that OMVs and antigens can establish sufficiently stable non-covalent interactions, which allows for the concomitant up-take of the adjuvant and antigen by the same antigen presenting cells. Such interactions depend on the physical-chemical properties of each antigen, and therefore vary from antigen to antigen. This explains why for some antigens (for example, Hla_H35L_), the OMV–antigen mixture is extremely efficient in eliciting elevated antibody titers, while for others (FhuD2), the absorption to the OMVs is inefficient, and therefore the mixture is poorly immunogenic. 

To circumvent the possible problem of inefficient antigen absorption to the OMVs, our work proposes two alternative solutions. The first one is to increase the amount of antigen to be added to the mixture. In general, the mixture of 1 μg of OMVs + 1 μg of antigen is enough to guarantee elevated antigen-specific antibody titers. The second one is the addition of alum, which, by promoting the formation of aggregates, always leads to the elicitation of saturating levels of antibody titers. Alum also appears to potentiate the already high antibody responses obtained with engineered OMVs alone (see Figure 5 and [25]), and seems to give a more balanced Th1/Th2 response, which can be advantageous for optimal protection against specific pathogens. 

OMV endogenous proteins are often considered an issue for the development of vaccines based on engineered OMVs for two main reasons. First, they are expected to negatively affect the immune responses against the heterologous antigens expressed in the OMVs. Second, the immune responses elicited by endogenous proteins raise safety concerns. Regarding the effect of endogenous proteins on the immunogenicity of heterologous antigens, we believe that, in fact, they provide a large repertoire of MHC II epitopes, which elicit a population of effector helper T cells capable of activating the B cells specific for the heterologous antigens. Such a contribution explains why even the OMV proteins present at relatively low concentrations are highly immunogenic.

As far as the safety issues are concerned, it is important to point out that it has been elegantly demonstrated that in humans, selective gut symbiotic Gram-negative bacteria including *E. coli* disseminate systemically and induce IgG responses that confer protection against infections by pathogenic bacteria such as Salmonella [42]. Therefore, anti-*E. coli* antibodies are not only naturally present in the human population, but are also beneficial in combating systemic infection through the induction of protective IgG responses. 

Taken together, we believe that *E. coli* OMVs have all the necessary features to become a promising platform to make safe and effective vaccines against infectious diseases. 

## Figures and Tables

**Table 2:** Immunogenicity and concentration of the selected OMV proteins.

Protein Name	Immunogenicity		Concentration (%)
	WB	ELISA	
OmpF	−/+	+	40
LpoA	+	+	8
MalE	+	+	2
SurA	+	+	2
BamA	+	+	1.5
YncE	+	+	1
CpoB	−/+	+	1
FhuE	+	+	1
FkpA	+	+	1
OppA	+	+	0.2
HisJ	−	−	0.1
GlpQ	−	+	0.02
RlpA	+	+	ND
YbiS	−	−	ND
YdcL	−/+	−	ND
Pal	−	+	ND

ND: not determined.

## Data Availability

The data that support the findings of this study are available from the corresponding author upon reasonable request.

## Supplementary Materials

The following supporting information can be downloaded at: https://www.mdpi.com/article/10.3390/membranes13110882/s1, Figure S1: SDS-PAGE analysis of the 16 purified OMVs proteins; Figure S2: SDS-PAGE analysis of the engineered OMVs expressing FhuD2, SpAKKAA, HlaH35L, SLO and spyCEP antigens; Figure S3: Antigen- specific IgG titers in sera from mice immunized with OMVsΔ60 expressing heterologous antigens; Figure S4: IgG titers in mice immunized with OMVsΔ60 expressing heterologous antigens or with OMVsΔ60 -antigen mixtures; Table S1: Main recombinant protein features exploited for the purification process; Table S2: Plasmid constructs used for the production of engineered OMVs and the expression of recombinant proteins; Table S3: List of primers.

## References

[1] Kulp A., Kuehn M.J. (2010). Biological Functions and Biogenesis of Secreted Bacterial Outer Membrane Vesicles. Annu. Rev. Microbiol..

[2] Ellis T.N., Kuehn M.J. (2010). Virulence and Immunomodulatory Roles of Bacterial Outer Membrane Vesicles. Microbiol. Mol. Biol. Rev..

[3] Moshiri A., Dashtbani-Roozbehani A., Peerayeh S.N., Siadat S.D. (2012). Outer membrane vesicle: A macromolecule with multifunctional activity. Hum. Vaccin. Immunother..

[4] Ellis T.N., Leiman S.A., Kuehn M.J. (2010). Naturally produced outer membrane vesicles from *Pseudomonas aeruginosa* elicit a potent innate immune response via combined sensing of both lipopolysaccharide and protein components. Infect. Immun..

[5] Kaparakis M., Turnbull L., Carneiro L., Firth S., Coleman H.A., Parkington H.C., Le Bourhis L., Karrar A., Viala J., Mak J. (2010). Bacterial membrane vesicles deliver peptidoglycan to NOD1 in epithelial cells. Cell Microbiol..

[6] Chen D.J., Osterrieder N., Metzger S.M., Buckles E., Doody A.M., DeLisa M.P., Putnam D. (2010). Delivery of foreign antigens by engineered outer membrane vesicle vaccines. Proc. Natl. Acad. Sci. USA.

[7] Kim O.Y., Hong B.S., Park K.-S., Yoon Y.J., Choi S.J., Lee W.H., Roh T.-Y., Lötvall J., Kim Y.-K., Gho Y.S. (2013). Immunization with *Escherichia coli* Outer Membrane Vesicles Protects Bacteria-Induced Lethality via Th1 and Th17 Cell Responses. J. Immunol..

[8] Kesty N.C., Kuehn M.J. (2004). Incorporation of Heterologous Outer Membrane and Periplasmic Proteins into *Escherichia coli* Outer Membrane Vesicles. J. Biol. Chem..

[9] Fantappiè L., de Santis M., Chiarot E., Carboni F., Bensi G., Jousson O., Margarit I., Grandi G. (2014). Antibody-mediated immunity induced by engineered *Escherichia coli* OMVs carrying heterologous antigens in their lumen. J. Extracell. Vesicles.

[10] Schroeder J., Aebischer T. (2009). Recombinant outer membrane vesicles to augment antigen-specific live vaccine responses. Vaccine.

[11] Fantappiè L., Irene C., De Santis M., Armini A., Gagliardi A., Tomasi M., Parri M., Cafardi V., Bonomi S., Ganfini L. (2017). Some gram-negative lipoproteins keep their surface topology when transplanted from one species to another and deliver foreign polypeptides to the bacterial surface. Mol. Cell. Proteom..

[12] Granoff D.M. (2010). Review of meningococcal group B vaccines. Clin. Infect. Dis..

[13] Ferrari G., Garaguso I., Adu-Bobie J., Doro F., Taddei A.R., Biolchi A., Brunelli B., Giuliani M.M., Pizza M., Norais N. (2006). Outer membrane vesicles from group B *Neisseria meningitidis* Δgna33 mutant: Proteomic and immunological comparison with detergent-derived outer membrane vesicles. Proteomics.

[14] Bernadac A., Gavioli M., Lazzaroni J.C., Raina S., Lloubès R. (1998). *Escherichia coli* tol-pal Mutants Form Outer Membrane Vesicles. J. Bacteriol..

[15] Deatherage B.L., Lara J.C., Bergsbaken T., Barrett S.L.R., Lara S., Cookson B.T. (2009). Biogenesis of bacterial membrane vesicles. Mol. Microbiol..

[16] McBroom A.J., Kuehn M.J. (2007). Release of outer membrane vesicles by Gram-negative bacteria is a novel envelope stress response. Mol. Microbiol..

[17] Scorza F.B., Colucci A.M., Maggiore L., Sanzone S., Rossi O., Ferlenghi I., Pesce I., Caboni M., Norais N., Di Cioccio V. (2012). High yield production process for Shigella outer membrane particles. PLoS ONE.

[18] Ladhani S.N., Ramsay M., Borrow R., Riordan A., Watson J.M., Pollard A.J. (2016). Enter B and W: Two new meningococcal vaccine programmes launched. Arch. Dis. Child..

[19] Serruto D., Bottomley M.J., Ram S., Giuliani M.M., Rappuoli R. (2012). The new multicomponent vaccine against meningococcal serogroup B, 4CMenB: Immunological, functional and structural characterization of the antigens. Vaccine.

[20] Gerke C., Colucci A.M., Giannelli C., Sanzone S., Vitali C.G., Sollai L., Rossi O., Martin L.B., Auerbach J., Di Cioccio V. (2015). Production of a *Shigella sonnei* vaccine based on generalized modules for membrane antigens (GMMA), 1790GAHB. PLoS ONE.

[21] Rossi O., Caboni M., Negrea A., Necchi F., Alfini R., Micoli F., Saul A., MacLennan C.A., Rondini S., Gerke C. (2016). Toll-Like Receptor Activation by Generalized Modules for Membrane Antigens from Lipid A Mutants of *Salmonella enterica* Serovars Typhimurium and Enteritidis. Clin. Vaccine Immunol..

[22] Orenstein W., Offit P.A., Edwards K.M., Plotkin S.A. (2017). Plotkin’s Vaccines.

[23] Huang W., Wang S., Yao Y., Xia Y., Yang X., Li K., Sun P., Liu C., Sun W., Bai H. (2016). Employing *Escherichia coli*-derived outer membrane vesicles as an antigen delivery platform elicits protective immunity against *Acinetobacter baumannii* infection. Sci. Rep..

[24] Kuipers K., Daleke-Schermerhorn M.H., Jong W.S., Hagen-Jongman C.M.T., van Opzeeland F., Simonetti E., Luirink J., de Jonge M.I. (2015). Salmonella outer membrane vesicles displaying high densities of pneumococcal antigen at the surface offer protection against colonization. Vaccine.

[25] Irene C., Fantappiè L., Caproni E., Zerbini F., Anesi A., Tomasi M., Zanella I., Stupia S., Prete S., Valensin S. (2019). Bacterial outer membrane vesicles engineered with lipidated antigens as a platform for *Staphylococcus aureus* vaccine. Proc. Natl. Acad. Sci. USA.

[26] Klock H.E., Lesley S.A. (2009). The Polymerase Incomplete Primer Extension (PIPE) method applied to high-throughput cloning and site-directed mutagenesis. Methods Mol. Biol..

[27] Zanella I., König E., Tomasi M., Gagliardi A., Frattini L., Fantappiè L., Irene C., Zerbini F., Caproni E., Isaac S.J. (2021). Proteome-minimized outer membrane vesicles from *Escherichia coli* as a generalized vaccine platform. J. Extracell. Vesicles.

[28] Kim O.Y., Park H.T., Dinh N.T.H., Choi S.J., Lee J., Kim J.H., Lee S.-W., Gho Y.S., Thi N. (2017). Bacterial outer membrane vesicles suppress tumor by interferon-γ-mediated antitumor response. Nat. Commun..

[29] Thoma J., Manioglu S., Kalbermatter D., Bosshart P.D., Fotiadis D., Müller D.J. (2018). Protein-enriched outer membrane vesicles as a native platform for outer membrane protein studies. Commun. Biol..

[30] Bhakdi S., Tranum-Jensen J. (1991). Alpha-Toxin of *Staphylococcus aureus*. Microbiol. Rev..

[31] Mariotti P., Malito E., Biancucci M., Lo Surdo P., Mishra R.P.N., Nardi-Dei V., Savino S., Nissum M., Spraggon G., Grandi G. (2013). Structural and functional characterization of the *Staphylococcus aureus* virulence factor and vaccine candidate FhuD2. Biochem. J..

[32] Falugi F., Kim H.K., Missiakas D.M., Schneewind O. (2013). Role of protein a in the evasion of host adaptive immune responses by *Staphylococcus aureus*. mBio.

[33] Weller U., Muller L., Messner M., Palmer M., Valeva A., Tranum-Jensen J., Agrawal P., Biermann C., Dobereiner A., Kehoe M.A. (1996). Expression of active streptolysin O in *Escherichia coli* as a maltose-binding-protein-streptolysin-O fusion protein. The N-terminal 70 amino acids are not required for hemolytic activity. Eur. J. Biochem..

[34] Zingaretti C., Falugi F., Nardi-Dei V., Pietrocola G., Mariani M., Liberatori1 Marilena Gallotta S., Tontini M., Tani C., Speziale P., Grandi G. (2010). *Streptococcus pyogenes* 95 SpyCEP: A chemokine-inactivating protease with unique structural and biochemical features. FASEB J..

[35] Xu Z., Moyle P.M. (2017). Bioconjugation Approaches to Producing Subunit Vaccines Composed of Protein or Peptide Antigens and Covalently Attached Toll-Like Receptor Ligands. Bioconjugate Chem..

[36] Cookson B.T., Alaniz R.C., Deatherage B.L., Lara J.C. (2022). Protective Immunity In Vivo and T Cell Responses, and Stimulate Potently Activate Dendritic Cells, Prime B That *Salmonella typhimurium* Facsimiles of Membrane Vesicles Are Immunogenic. J. Immunol. Ref..

[37] Adriani R., Gargari S.L.M., Nazarian S., Sarvary S., Noroozi N. (2018). Immunogenicity of Vibrio cholerae outer membrane vesicles secreted at various environmental conditions. Vaccine.

[38] Qasim M., Wrage M., Nüse B., Mattner J. (2022). Shigella Outer Membrane Vesicles as Promising Targets for Vaccination. Int. J. Mol. Sci..

[39] Acevedo R., Fernã¡Ndez S., Zayas C., Acosta A., Sarmiento M.E., Ferro V.A., Rosenqvist E., Campa C., Cardoso D., Garcia L. (2014). Bacterial outer membrane vesicles and vaccine applications. Front. Immunol..

[40] Bai X., Findlow J., Borrow R. (2011). Recombinant protein meningococcal serogroup B vaccine combined with outer membrane vesicles. Expert Opin. Biol. Ther..

[41] Cheng K., Zhao R., Li Y., Qi Y., Wang Y., Zhang Y., Qin H., Qin Y., Chen L., Li C. (2021). Bioengineered bacteria-derived outer membrane vesicles as a versatile antigen display platform for tumor vaccination via Plug-and-Display technology. Nat. Commun..

[42] Zeng M.Y., Cisalpino D., Varadarajan S., Hellman J., Warren H.S., Cascalho M., Inohara N., Núñez G. (2016). Gut Microbiota-Induced Immunoglobulin G Controls Systemic Infection by Symbiotic Bacteria and Pathogens. Immunity.

