# Supplementary material for: Immunogenicity of Escherichia coli Outer Membrane Vesicles: Elucidation of Humoral Responses against OMV-Associated Antigens
Source: Membranes (Basel). 2023 Nov 16;13(11):882. doi: 10.3390/membranes13110882 (PMC10673343; doi:10.3390/membranes13110882)
Supplement: Supplementary file 1 [file membranes-13-00882-s001.zip › membranes-2675546-supplementary.pdf]

Immunogenicity of *Escherichia coli* Outer Membrane Vesicles: Elucidation of Humoral Responses against OMV-Associated Antigens

Lorenzo Croia [1], Giulia Boscato Sopetto [1], Ilaria Zanella [1], Elena Caproni [2], Assunta Gagliardi [2], Silvia Tamburini [2], Enrico Koenig [1], Mattia Benedet [2], Gabriele Di Lascio [2], Riccardo Corbellari [1], Alberto Grandi [2,3], Michele Tomasi [1] and Guido Grandi [1,*]

[1] Department of Cellular, Computational and Integrative Biology (CIBIO), University of Trento, Via Sommarive 9, 38123 Trento, Italy; lorenzo.croia@unitn.it (L.C.); g.boscatosopetto@unitn.it (G.B.S.); ilaria.zanella@unitn.it (I.Z.); koenig.enrico@gmail.com (E.K.); riccardo.corbellari@unitn.it (R.C.); michele.tomasi.2@unitn.it (M.T.)

[2] Toscana Life Sciences Foundation, Via Fiorentina 1, 53100 Siena, Italy; e.caproni@toscanalifesciences.org (E.C.); a.gagliardi@toscanalifesciences.org (A.G.); silvia.tamburini@unitn.it (S.T.); m.benedet@toscanalifesciences.org (M.B.); g.dilascio@toscanalifesciences.org (G.D.L.); a.grandi@toscanalifesciences.org (A.G.)

[3] BiOMViS Srl, Via Fiorentina 1, 53100 Siena, Italy

* Correspondence: guido.grandi@unitn.it

Supplementary Materials

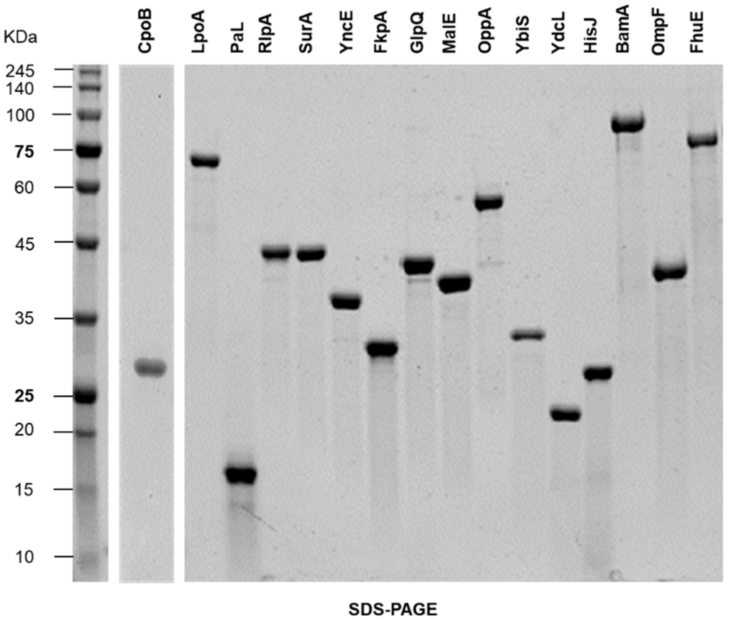

Figure S1. SDS-PAGE analysis of the 16 purified OMVs proteins. The 16 selected proteins were cloned in the pET15b plasmid as N-terminal TEV-His-tag fusion proteins and expressed in the *E. coli* BL21(DE3) producer strain. Proteins were purified by IMAC, TEV digested, and SEC. Two μg of purified recombinant proteins was loaded on SDS-PAGE and revealed by Coomassie staining. The correctness of the protein purification process was verified by matching protein bands with the correspondent predicted molecular weight.

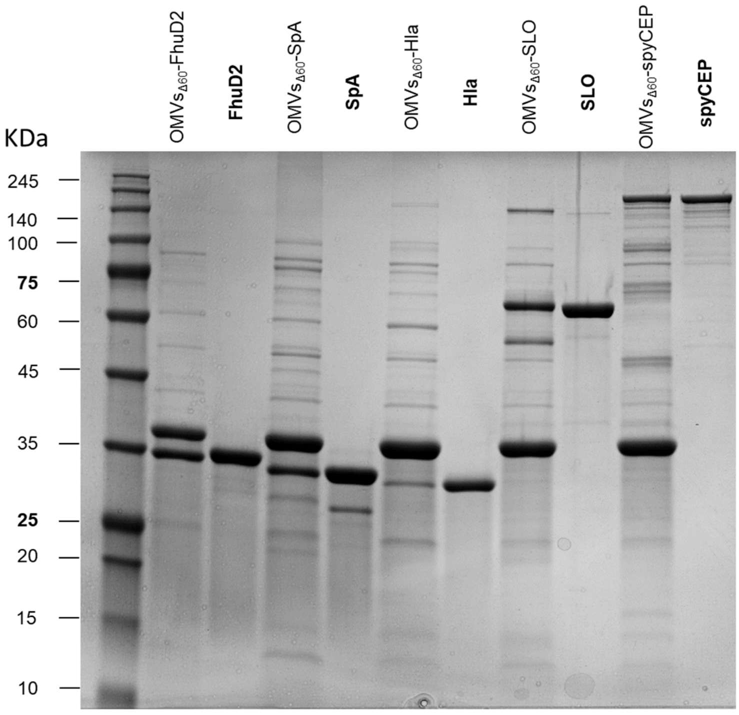

Figure S2. SDS-PAGE analysis of the engineered OMVs expressing the FhuD2, SpA_{KKAA}, Hla_{H35L}, SLO, and spyCEP antigens. The corresponding purified recombinant proteins were run next to each engineered OMV.

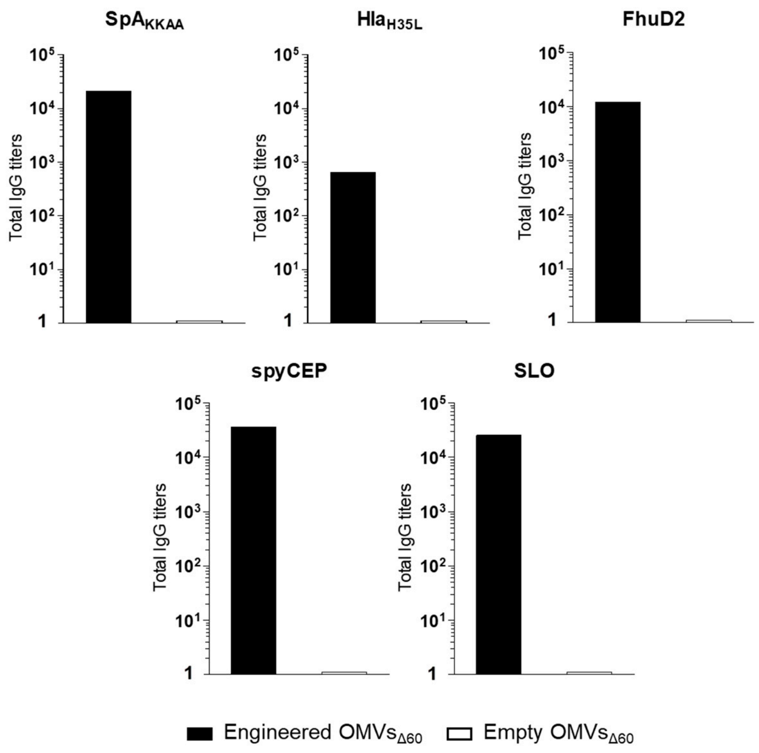

Figure S3. Antigen- specific IgG titers in sera from mice immunized with $OMVs_{\Delta 60}$ expressing heterologous antigens.

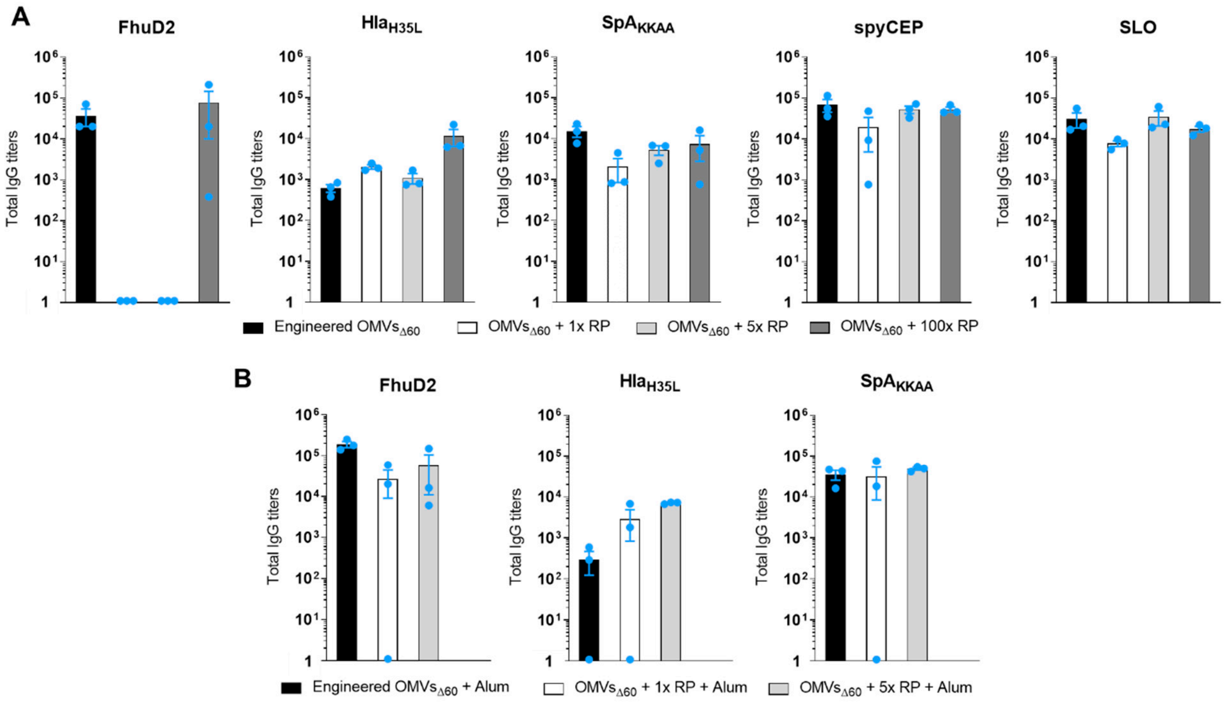

Figure S4. **IgG titers in mice immunized with $OMVs_{\Delta 60}$ expressing heterologous antigens or with $OMVs_{\Delta 60}$ –antigen mixtures.** (**A**) Groups of CD1 female mice (four per group) were i.p. immunized three times at 2-week intervals with 1 µg of engineered $OMVs_{\Delta 60}$ expressing FhuD2, SpAKKAA, HlaH35L, SLO, or spyCEP or with 1 µg $OMVs_{\Delta 60}$ mixed with 1x, 5x, and 100x the amounts of antigens present in the engineered OMVs. Ten days after the last immunization, sera were collected and IgG titers in single sera from three mice of each group

were analyzed by ELISA using plates coated with the corresponding recombinant antigens. (**B**) The same immunization schedule was performed in the presence of alum, which was added to 1 µg of the engineered OMVs expressing the FhuD2, SpAKKAA, or HlaH35L antigens and to 1 µg $OMVs_{\Delta 60}$ + 5x the amount of antigens present in the engineered OMVs. The antigen-specific ELISA titers were determined as described above. RP, recombinant protein. Error bar represents the standard error of the mean (S.E.M.).

Table S1. Main recombinant protein features exploited for the purification process.

Protein	Compartment	Solubility	MW [kDa]	pI	Molar extinction coefficient (Ɛ)	Buffer A and B pH
BamA	OM	Insoluble	88,4	4.9	140165	7.6
CpoB	PP	Soluble	25.4	8	21890	7.6
FhuE	OM	Insoluble	77.4	4.5	143590	7.6
FkpA	PP	Soluble	26.2	6.7	15930	7.6
GlpQ	PP	Soluble	38.2	5.2	51800	7.2
HisJ	PP	Soluble	26.2	5.2	17545	7.6
LpoA	LP	Soluble	70	5.1	67505	7.6
MalE	PP	Soluble	40.7	5.2	66350	7.2
OmpF	OM	Insoluble	37	4.6	54210	7.6
OppA	PP	Soluble (partially)	58.4	6.0	113345	7.2
Pal	LP	Soluble	16.6	6.1	11920	7.6
RlpA	LP	Soluble	35.7	5.1	16055	7.0
SurA	PP	Soluble	45	6.1	29450	7.6
YbiS	PP	Soluble (partially)	30,8	5.6	27390	7.6
YdcL	LP	Soluble	22.4	6.8	22920	7.6
YncE	PP	Soluble	35.3	8.8	28420	7.0
Hla_{H35L}	-	Soluble	30	8.2	64860	6.3
FhuD2	-	Soluble	34	9.7	55350	7.6
SpA_{KKAA}	-	Soluble	28.3	4.6	5960	7.6
SLO	-	Soluble	63.6	6.0	71280	7.5
SpyCEP	-	Soluble	180.7	6.7	151850	7.5

The table summarizes the recombinant protein features used in this work. Information on protein compartmentalization was retrieved from UniProt. Protein solubility was evaluated with the B-PER® protein solubility test. Molar extinction coefficient, isoelectric point (pI), and molecular weight were predicted with the EMBOSS-Pepstats tool (EMBL-EBI). The pH at which protein purification was carried out is reported accordingly.

Table S2. Plasmid constructs used for the production of the engineered OMVs and the expression of recombinant proteins.

Plasmid name	Source
**Plasmids for production of engineered OMVs**	
pET21b(+)_lpp_FhuD2	[25]
pET21b(+)_lpp_Hla$_{H35L}$	[25]
pET21b(+)_lpp_SpA$_{KKAA}$	[25]
pET21b(+)_lpp_SLO	BiOMViS
pACYC_lpp_spyCEP	BiOMViS
**Plasmids for expression of *E. coli* recombinant proteins**	
pET15b_His6_TEV_BamA	This work
pET15b_His6_TEV_CpoB	[34]
pET15b_His6_TEV_FhuE	This work
pET15b_His6_TEV_FkpA	[34]
pET15b_His6_TEV_GlpQ	[34]
pET15b_His6_TEV_HisJ	This work
pET15b_His6_TEV_LpoA	This work
pET15b_His6_TEV_MalE	[34]
pET15b_His6_TEV_OmpF	This work
pET15b_His6_TEV_OppA	[34]
pET15b_His6_TEV_PaL	[34]
pET15b_His6_TEV_RlpA	This work
pET15b_His6_TEV_SurA	This work
pET15b_His6_TEV_YbiS	This work
pET15b_His6_TEV_YdcL	This work
pET15b_His6_TEV_YncE	This work
**Plasmids for expression of *S.aureus* recombinant antigens**	
pET15b_His6_TEV_FhuD2	[25]
pET15b_His6_TEV_Hla$_{H35L}$	[25]
pET15b_His6_TEV_ SpA$_{KKAA}$	[25]
**Plasmids for expression of GAS recombinant antigens**	
pET15b_His6_TEV_SLO	This work
pET15b_His6_TEV_spyCEP	This work

Table S3. List of primers.

Code	Description	Sequence (5′-3′)
**Primers for Vector (V) PIPE PCR**		
EK-1084	Primer forward V-PIPE pET21b	GCCCTGGAAGTACAGGTTTTC
EK-1085	Primer reverse V-PIPE pET21b	CGCGACTTAATTCTAGCATAAC
Pacyc-F	Primer forward V-PIPE pACYC	AGCCAGGATCCGAATTCGAGC
Lpp-Cys-R	Primer reverse V-PIPE pACYC	GCTGGAGCAACCTGCCAGCAGAG
**Primers for Insert (I) PIPE-PCR ("flap" primer)**		
EK-1486	Primer forward I-PIPE BamA	ctgtacttccagggc GCTGAAGGGTTCGTAGTGAA
LC-1498	Primer reverse I-PIPE BamA	tagaattaagtcgcg TTACCAGGTTTTACCGATGTTAAAC
i-f-cpoB	Primer forward I-PIPE CpoB	ctgtacttccagggc CAGGCACCAATCAGTA
i-r-cpoB	Primer reverse I-PIPE CpoB	tagaattaagtcgcg TTACATCGCGTTCAGACGT
EK-1486	Primer forward I-PIPE FhuE	ctgtacttccagggc GCACCAGCCACTGAAGAAAC
LC-1499	Primer reverse I-PIPE FhuE	tagaattaagtcgcg TCAGAATTGATACGTGCCGGTAA
i-f-fkpA	Primer forward I-PIPE FkpA	ctgtacttccagggc ATGAAATCACTGTTTAAAGTAACGC
i-r-fkpA	Primer reverse I-PIPE FkpA	tagaattaagtcgcg TTATTTTTTAGCAGAATCTGCGGC
i-f-glpQ	Primer forward I-PIPE GlpQ	ctgtacttccagggc ATGAAATTGACGCTGAAAAACC
i-r-glpQ	Primer reverse I-PIPE GlpQ	tagaattaagtcgcg TTACTCTTTATTAAGGAATTTTACTGCC
EK-1482	Primer forward I-PIPE HisJ	ctgtacttccagggc GCGATTCCGCAAAACATCCG
LC-1494	Primer reverse I-PIPE HisJ	tagaattaagtcgcg TTAGCCACCATAAACATCAAAATCGAAG
EK-1484	Primer forward I-PIPE LpoA	ctgtacttccagggc TGTGGCACCCATACTCCCGA
LC-1496	Primer reverse I-PIPE LpoA	tagaattaagtcgcg TTAACTGACGGGGACTACCTGACC
i-f-malE	Primer forward I-PIPE MalE	ctgtacttccagggc ATGAAAATAAAAACAGGTGCACGC
i-r-malE	Primer reverse I-PIPE MalE	tagaattaagtcgcg TTACTTGGTGATACGAGTCTGC
EK-1479	Primer forward I-PIPE OmpF	ctgtacttccagggc GCAGAAATCTATAACAAAGATGGCA
LC-1491	Primer reverse I-PIPE OmpF	tagaattaagtcgcg TTAGAACTGGTAAACGATACCCAC
i-f-oppA	Primer forward I-PIPE OppA	ctgtacttccagggc ATGACCAACATCACCAAGAGAAG
i-r-oppA	Primer reverse I-PIPE OppA	tagaattaagtcgcg TTAGTGCTTCACAATGTACATATTCCG
i-f-pal	Primer forward I-PIPE PaL	ctgtacttccagggc TGTTCTTCCAACAAGAA

i-r-pal	Primer reverse I-PIPE PaL	tagaattaagtcgcg TTAGTAAACCAGTACCGCA
EK-1485	Primer forward I-PIPE RlpA	ctgtacttccagggc TGTACAAGCGATGATGGTCAG
LC-1497	Primer reverse I-PIPE RlpA	tagaattaagtcgcg CTACTGCGCGGTAGTAATAAATGA
EK-1489	Primer forward I-PIPE SurA	ctgtacttccagggc GCCCCCCAGGTAGTCGATAA
LC-1501	Primer reverse I-PIPE SurA	tagaattaagtcgcg TTAGTTGCTCAGGATTTTAACGTAGGC
EK-1483	Primer forward I-PIPE YbiS	ctgtacttccagggc GTAACTTATCCTCTGCCAACCG
LC-1495	Primer reverse I-PIPE YbiS	tagaattaagtcgcg TTAATTCAGACGAACCGGCATC
i-f-ydcL	Primer forward I-PIPE YdcL	ctgtacttccagggc ATGCGTACCACATCATTTGC
i-r-ydcL	Primer reverse I-PIPE YdcL	tagaattaagtcgcg CTACTTTTTGTTAACGTCAAACATGG
EK-1490	Primer forward I-PIPE YncE	ctgtacttccagggc GCAGAAGAAATGCTGCGTAAAG
LC-1502	Primer reverse I-PIPE YncE	tagaattaagtcgcg TTACAGCGCAATACGAATCACAT
EK-1051	Primer forward I-PIPE SLO	ctgtacttccagggc AACAAACAAAACACTGCTAGTA
EK-1052	Primer reverse I-PIPE SLO	tagaattaagtcgcg TTCGATTACTTATAAGTAGTAA
EK-1053	Primer forward I-PIPE spyCEP	ctgtacttccagggc ACTCAAAGAGCTCTTAAAGCA
EK-1054	Primer reverse I-PIPE spyCEP	tagaattaagtcgcg ATGGACTTAGAACAAACGAAG
**Primers for screenings**		
LC-1507	Primer reverse colony PCR	CTGAATACTGATTTTCTGTG
CI-1004	(T7P) Primer forward sequencing	CCCTATAGTGAGTCGTATTA
CI-1005	(T7T) Primer reverse sequencing	GCTAGTTATTGCTCAGCGG
